# Doped metal clusters as bimetallic AuCo nanocatalysts: insights into structural dynamics and correlation with catalytic activity by *in situ* spectroscopy†

**DOI:** 10.1039/d2fd00120a

**Published:** 2022-07-13

**Authors:** Noelia Barrabés, Jon Ostolaza, Sarah Reindl, Martin Mähr, Florian Schrenk, Hedda Drexler, Christoph Rameshan, Wojciech Olszewski, Günther Rupprechter

**Affiliations:** a Institute of Materials Chemistry, Technische Universität Wien Getreidemarkt 9/165 1060 Vienna Austria noelia.rabanal@tuwien.ac.at; b Department of Chemical Engineering and Biotechnology, University of Cambridge Philippa Fawcett Drive Cambridge CB3 0AS UK; c Faculty of Physics, University of Bialystok ul. K. Ciolkowskiego 1L 15-245 Bialystok Poland

## Abstract

Co-doped Au_25_ nanoclusters with different numbers of doping atoms were synthesized and supported on CeO_2_. The catalytic properties were studied in the CO oxidation reaction. In all cases, an enhancement in catalytic activity was observed compared to the pure Au_25_ nanocluster catalyst. Interestingly, a different catalytic performance was obtained depending on the number of Co atoms within the cluster. This was related to the mobility of atoms within the cluster’s structure under pretreatment and reaction conditions, resulting in active CoAu nanoalloy sites. The evolution of the doped Au clusters into nanoalloys with well-distributed Co atoms within the Au cluster structure was revealed by combined XAFS, DRIFTS, and XPS studies. Overall, these studies contribute to a better understanding of the dynamics of doped nanoclusters on supports upon pretreatment and reaction, which is key information for the future development and application of bimetallic nanocluster (nanoalloy) catalysts.

## Abstract 2

Cobalt doping of Au_25_ nanoclusters led to an enhancement in the catalytic behaviour. The evolution of the doped Au clusters to nanoalloys with controlled surface sites was revealed by combined XAFS, DRIFTS and XPS studies.

## Graphical abstract

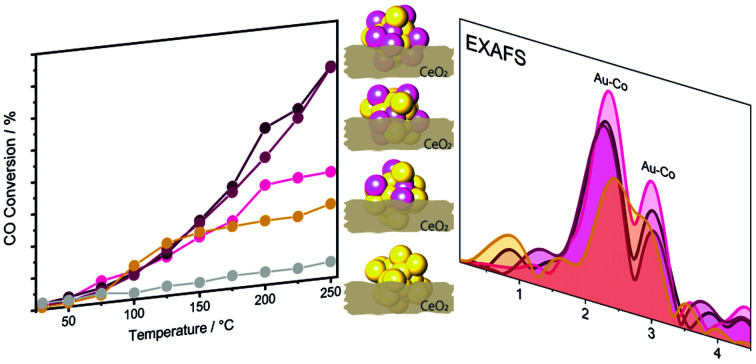

## Introduction

In nanocatalysis, a great challenge is to obtain truly homogenous and well-defined highly active nanostructures on surfaces. Ligand-protected metal nanoclusters are an emerging class of functional nanomaterials with atomic precision, well-defined molecular structure, and intriguing molecular-like properties. The size control during cluster synthesis opens new opportunities for accurate studies of size-dependent properties, atomic structure effects, and reaction mechanisms in catalysis.^1–5^ Thus, studies of heterogeneous catalysis *via* atomically-precise gold nanoclusters is an emerging field.^6–8^ Such well-defined nanocatalysts represent a rather new model system enabling fundamental insights into catalytic reactions.

The physical–chemical properties of gold nanoclusters can be fine-tuned by heteroatom doping, which has a strong influence on their reactivity and stability (towards thermal and/or chemical treatments for instance).^9–11^ Knowledge of the number of incorporated dopant atoms, their exact location in the cluster, as well as structure–property relationships are required for a thorough understanding. Depending on the nature of the dopant atom, different positions within the Au cluster have been identified, *e.g.* in the center (Pd, Pt, Cd), in the outer core shell (Ag, Cd) or in the protecting Au(i)–thiolate staple motifs (Cu, Hg) surrounding the core.^12,13^

In our previous work with doped systems, we were able to determine the exact position of two Pd atoms in the Pd_2_Au_36_(SR)_24_ cluster using X-ray absorption fine structure spectroscopy (XAFS).^14^ Recently, different Pd positions as a function of the number of doped atoms in Au_25_ nanoclusters and their evolution under reaction conditions were studied by operando spectroscopy.^10^ It was found that the bimetallic cluster strongly enhances CO conversion, which seems to be related to the migration of Pd atoms from the cluster center to the outer surface, resulting in a PdAu alloy with isolated Pd positions. Similar behavior was observed in Ag-doped Au_25_ nanoclusters on ITQ2 zeolite, leading to the formation of bimetallic AgAu sites that exhibit higher catalytic activity for CO oxidation than the monometallic centers. As these nanoalloys are formed by rearrangement of metal atoms during pretreatment and reaction, and due to the defined structure of the clusters, it is possible to understand their evolution using spectroscopic techniques.^9^

Another interesting bimetallic system is Co–Au, since the physicochemical properties of both metals complement each other and enhance, among others, the magnetic,^15,16^ catalytic^17,18^ or electrocatalytic^19,20^ properties. Moreover, the synergy of the biocompatibility of Au and the magnetic properties of Co expands its applicability to biomedicine and biodiagnostics.^21,22^ A challenge is the high oxidation sensitivity of cobalt, which becomes more evident as the particle size decreases.^15^

Several approaches have been explored for the preparation of Co–Au nanoalloys, including chemical synthesis,^15,23^ evaporation,^24^ or laser ablation.^16^ However, these methods result in the formation of nanoparticles with more than 100 atoms, where the plasmonic properties of gold also play a role. Therefore, we investigated the synthesis of Co–Au nanoalloys at the cluster level. In the present work, Co-doped Au_25_ nanoclusters were synthesized based on previous experience with the synthesis of bimetallic gold nanoclusters, obtaining three different fractions with different solubility and optical activity. Due to difficulties in mass spectrometric studies, we used XAFS spectroscopy to understand their differences. The catalytic properties in the CO oxidation were studied once supported on CeO_2_. An enhancement in catalytic behaviour was observed compared to the pure Au_25_ nanocluster catalyst. Interestingly, a different catalytic performance was obtained depending on the expected different number of Co atoms and positions within the cluster. This could be related to the atom mobility within the cluster structure upon pretreatment and reaction conditions, leading to a different configuration of the nanoalloy, as indicated both by the XAFS and infrared studies.

## Experimental

### Nanocluster synthesis and characterization

Co_*x*_Au_25−*x*_(SC_2_H_4_Ph)_18_ nanoclusters (named Co_*x*_Au_25−*x*_) were prepared by a modified protocol based on previous experience and reported methods.^10^ Un-doped Au_25_(SC_2_H_4_Ph)_18_ nanoclusters were prepared as a reference (named Au_25_). From the final black solid obtained in the bimetallic nanocluster synthesis, three consecutive extractions were performed with Acetonitrile (CoAuAcetonitrile), with Acetone (CoAuAcetone) and the rest with DCM (CoAuDCM). The three extractions were further purified by size exclusion chromatography. The nanoclusters were characterized by UV-Vis spectroscopy and Matrix-assisted Laser Desorption Ionization Mass Spectrometry (MALDI-MS). The supported catalysts were prepared by wet impregnation of the clusters on CeO_2_. The gold loading of the catalysts was 1.2 wt%. Further details on the protocols and characterization can be found in the ESI.†

### Kinetic studies of the CO oxidation reaction

Catalytic activity studies of the nanocluster catalysts in the CO oxidation reaction were performed using a flow reactor coupled to a micro gas chromatograph (Micro-GC, Fusion 3000A, Inficon). ∼15 mg of catalyst were used for all experiments. All catalysts were pretreated in an oxidative atmosphere (5% O_2_ in Ar, total gas flow of 50 ml min^−1^) with a temperature ramp of 10 °C min^−1^ up to 250 °C. The maximum temperature was maintained for 30 minutes before cooling the sample to room temperature in Ar (50 ml min^−1^). The gas flow composition was then changed to reaction conditions (1% CO and 2% O_2_ in Ar; 50 ml min^−1^ total gas flow). The temperature was increased to 250 °C with a ramp of 5 °C min^−1^ and was kept there for 30 min. After the reaction, the catalyst was cooled to room temperature under Argon.

### Infrared studies

*In situ*/*Operando* transmission Fourier-transform infrared studies (transmission FTIR) were conducted using a Bruker Vertex 70 spectrometer. The catalyst (around 10 mg) was ground thoroughly and pressed into a thin pellet using a hydraulic press. The pellet was then mounted in a flow cell with IR transmissible windows and a thermocouple connected to a PID controller. The product gas flow was analyzed by mass spectrometry (Pfeiffer Vacuum, Thermostar). The sample was pretreated as described before while simultaneously recording the IR spectra (MIR, resolution 4 cm^−1^). After cooling down to room temperature under helium, a CO adsorption experiment was performed. For this, the sample was exposed to 1% CO in He (50 ml min^−1^ total flow rate) until the IR band of CO stopped changing significantly. Subsequently, 50 ml min^−1^ of He was passed through the cell until no further significant changes in the IR spectrum were observed. Following the CO adsorption experiment, the CO oxidation reaction was performed and was monitored using IR. The sample was then cooled down to room temperature under He and the CO adsorption experiment was repeated.

### *Operando*/*in situ* XAFS studies

X-ray Absorption Fine Structure Spectroscopy (XAFS) measurements were performed at the CLAESS Beamline at the Alba Synchrotron in fluorescence mode (Co K-edge and Au-L_3_ edge) in the beamline's solid/gas reactor multipurpose cell. The catalysts were pressed into pellets. The samples were pretreated inside the multipurpose cell at 250 °C for 40 min under oxygen flow (pretO_2_; 5% O_2_ in He, total flow: 45 ml min^−1^) and cool down in He. After cooling down, the gas mixture was changed to reaction conditions (reaction: 1.7% CO, 3.3% O_2_ in He, total flow: 45 ml min^−1^). The samples were heated to 250 °C with a ramp of 5 °C min^−1^. The maximum temperature was held for 30 min, and then the reaction chamber was cooled down to RT under He flow. Extended X-ray Absorption Fine Structure (EXAFS) spectra were taken at 40 °C in He at the beginning, after pretreatment and after reaction for each sample, without opening the reaction chamber in between. The Artemis package^25^ that uses the FEFF8 code^26^ was applied for EXAFS data treatment and is described in the ESI.†

## Results and discussion

### Co doped Au_25_ nanoclusters

**Fig. 1:**
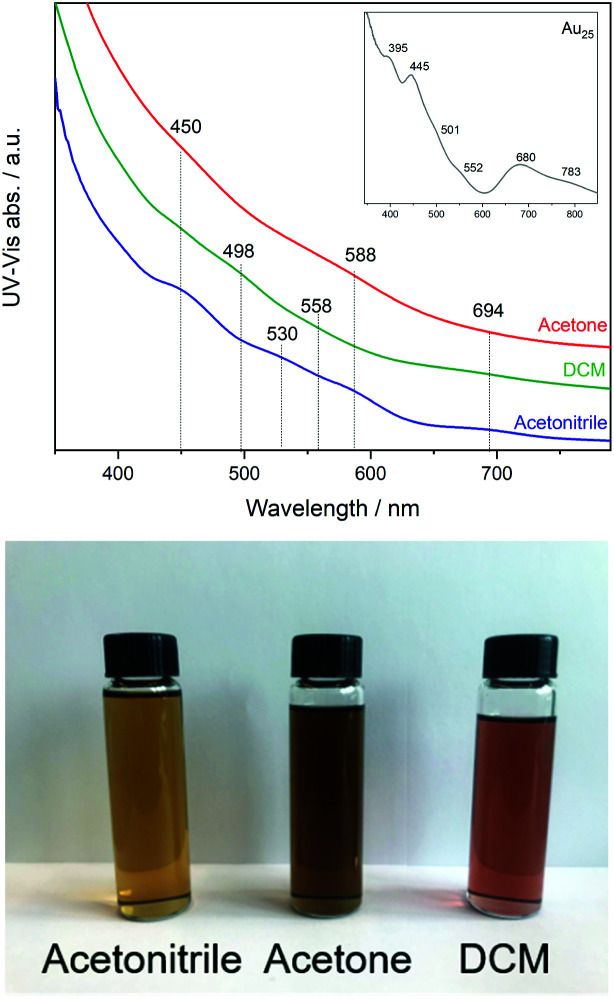
UV-Vis spectra of Co doped Au_25_ nanoclusters (inset spectrum of Au_25_ as reference) and photograph of the three solutions.

First, the three fractions obtained during synthesis were studied by UV-Vis revealing different optical activity. In Fig. 1, a broadening of the peaks and a shift compared to the spectra of the undoped Au_25_ clusters can be seen (inset Fig. 1).

This may be related to the substitution of Au atoms at different positions, which has been observed in previous studies of doped clusters.^27–29^ In the case of the shift to lower energy, the substitution at the Au staple position was associated with this, while the broadening of the peaks and the shift to higher energy were associated with the substitution of the Au atoms at the core surface.^27^

Due to difficulties obtaining MALDI-MS or Electrospray ionization (ESI) mass spectrometry to determine the exact composition of the three extracted fractions, complementary studies by XAFS were performed to gain insight into the possible structures and compositions of the clusters. The samples were analyzed at the Co K-edge and the Au L_3_-edge. The low content of cobalt atoms in the Au nanocluster resulted in difficulties in obtaining high quality data at the Co–K edge, as can be seen in Fig. S2,† so no clear interpretation is possible.

The significant change in absorption in the XANES spectrum occurs at the absorption edge formed by transitions of electrons between occupied nuclear states and unoccupied valence states. Due to the transitions of electrons, the absorption edge is sensitive to the effective nuclear charge. For this reason, the absorption edge can help in the study of electronic properties such as oxidation. The rising absorption edge can lead to a sharp, intense peak called the “white line”, which is related to the transport of excited core electrons to unoccupied valence levels *via* a dipole-allowed transition. The intensity of the white line can therefore be used to quantitatively or qualitatively determine the electronic structure of the valence level of the absorbing element. The intensity of the white line increases from bulk to cluster structure, indicating a depletion of d electrons with decreasing size.

**Fig. 2:**
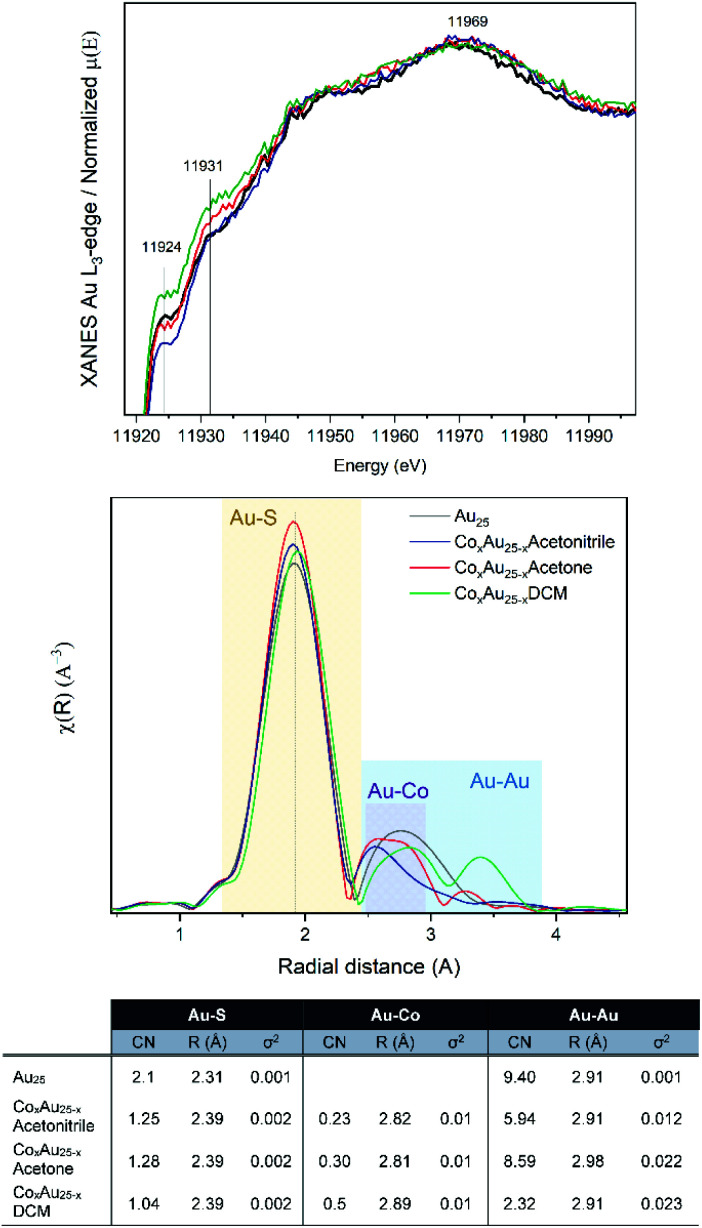
XAFS results at the Au L_3_-edge of AuCo nanoclusters: (from top) XANES, *R*-space and EXAFS fitting.

Based on the small energy edge jump and position, one can estimate a low content of cobalt atoms within the Au cluster, which may be bound to Au and S. Therefore, the samples were studied at the Au-L_3_ edge, with the XANES and EXAFS analysis shown in Fig. 2. The Co-doped clusters are compared with undoped Au_25_ nanoclusters. In all cases, differences in the intensity of the white line (11 924 eV) are observed, which can be attributed to the presence of unoccupied 5d states (d-holes). The intensity of the white line can be related to the size and charge transfer. Therefore, a lower intensity can be associated with smaller particles or with fewer vacancies in the 5d band related to the interaction with Co. Upon closer inspection, the CoAuAcetone sample shows no differences from the pure Au_25_ cluster, in contrast to CoAuAcetonitrile (lower white line) and CoAuDCM (higher white line).

From the *R*-space analysis, the peak related to the Au–S bonds is clearly around 2.0 Å. However, the distance between the Au–Co bonds is generally between 2.5 and 2.8 Å, which makes their distinction from the Au–Au peaks quite difficult. The EXAFS fit considering the Au_25_ structure allows us to estimate the different Au–Co compositions of the three samples as a function of the coordination numbers (CN).

### Catalytic activity of bimetallic cluster catalyst

**Fig. 3:**
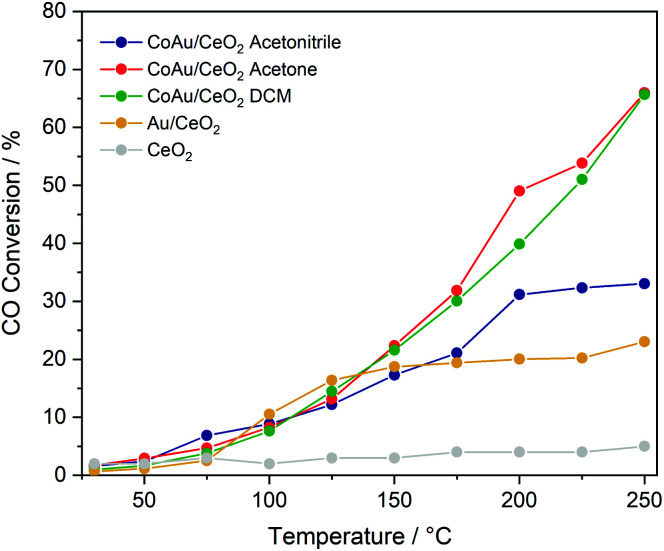
Catalytic activity of nanocluster catalysts in CO oxidation.

To evaluate the effect of doping on the reactivity of the nanoclusters in the CO oxidation reaction, the three CoAu nanoclusters and the undoped Au_25_ were supported on CeO_2_. While in the lower temperature range all catalysts show the same tendency, significant differences are observed above 175 °C (Fig. 3). CoAuAcetone and DCM catalysts show higher activity, which increases with temperature, in contrast to CoAuAcetonitrile and Au_25_, which an activity plateau at 200 °C.

### Infrared studies

In order to explain the differences in catalytic activity and to correlate them with the possible structural dynamics of the bimetallic clusters, previously observed for Pd^10^ or Ag^9^ doped Au_25_ catalysts under reaction conditions, *in situ* IR studies were performed. Fig. S3† shows the temperature dependent infrared spectra of the three catalyst under CO oxidation reaction conditions (as in the kinetic studies). Catalytic activity is indicated by the formation of CO_2_ gas phase bands between 2400 and 2300 cm^−1^ and the decrease of the CO gas phase bands between 2100 and 2200 cm^−1^. With increasing temperature, a shift is observed in the CO region related to the contribution of CO Au bands.

**Fig. 4:**
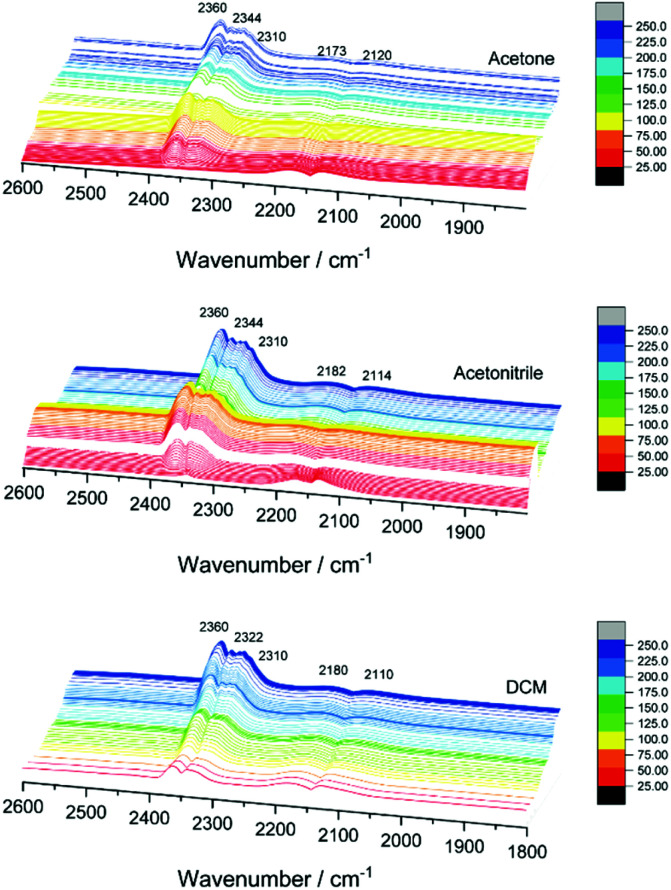
*In situ* infrared spectra of CO oxidation on AuCo/CeO_2_ catalysts.

In the CO_2_ region, several bands evolved between 2305 and 2350 cm^−1^ when increasing the temperature (Fig. 4), which was not observed in previous studies using monometallic or other bimetallic nanoclusters on CeO_2_ catalysts in CO oxidation.^9,10,30^ CO_2_ adsorption on cobalt oxide may occur at 2350 cm^−1^, but mainly at room temperature, with bands decreasing with increasing temperature and disappearing above 100 °C, and neither were detected in the XAFS or XPS results.^31^ Therefore, the 2310, 2322 or 2344 cm^−1^ bands obtained at higher temperatures may be related to the strong interaction of CO_2_ with metallic Co on the gold nanocluster or bimetallic (alloyed) CoAu sites. The strong interaction of CO_2_ with other metals and materials at high temperatures, leading to infrared bands in this region, has been reported earlier.^32,33^

**Fig. 5:**
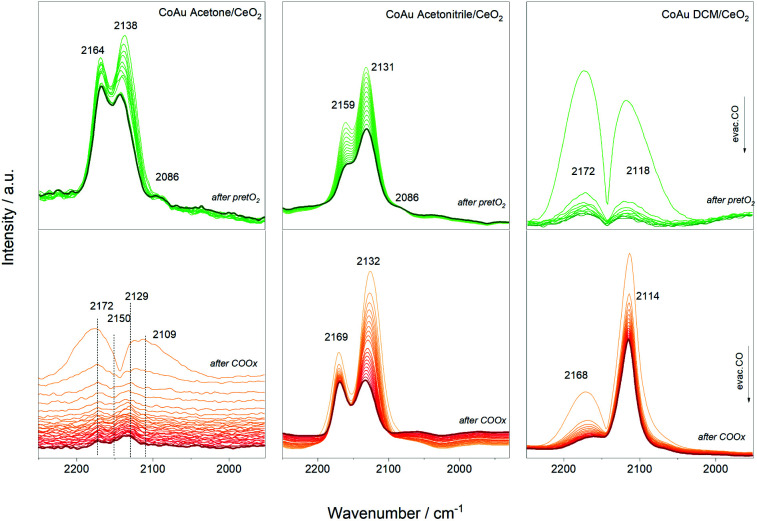
Infrared spectra during evacuation after CO dosing experiments.

Furthermore, CO dosing experiments were performed with the samples before (after pretO_2_) and after CO oxidation (after COox) steps. Fig. 5 shows the spectral evolution during evacuation of the CO atmosphere. The bands related to CO–Co are expected around 1940–1970 cm^−1^ in the case of bridge CO adsorbed on Co^0^, around 2030–2025 cm^−1^ for linearly adsorbed CO on metallic cobalt, 2070–2110 cm^−1^ for Co^+^, 2120–2170 cm^−1^ for Co^2+^, and 2178–2180 cm^−1^ for Co^3+^.^34^ The Co^+^/Co^2+^ sites can be related to cobalt interacting with another metal as observed for other cobalt-based materials.^35,36^ In addition, the bands related to CO–Au are expected between 2070–1950 cm^−1^ due to Au^0^ (around 2098 cm^−1^) and Au^*δ*+^ (2125 cm^−1^) species.

The evolution of the three catalysts is different after the oxidative pretreatment which could be related to the different content of cobalt atoms and their location inside the cluster structure. In the case of the CoAu acetone and acetonitrile samples, three main bands around 2160 cm^−1^, 2130 cm^−1^ and 2086 cm^−1^ appeared which could be assigned to CO–Co^+^, CO–Au^*δ*+^ and CO–Au^0^, respectively, whereas in case of DCM mainly CO–Co^+^ and CO–Au^0^ are observed. This is consistent with the XPS results showing different oxidation states of the gold in the acetonitrile samples, while only metallic states are seen in the DCM one (Fig. S4†). A similar trend is denoted after reaction, with different intensity relations between the bands. The strong presence of CO–Au^*δ*+^ and CO–Co^+^ may indicate Co–Au alloy particle formation as observed before with doped cluster catalysts.^9,10,27–29,37^ Previous studies reported that the interfacial contact between Au and CoO_*x*_ resulted in a highly reducible and active CoO_*x*_ phase that worked in synergy with the Au particles.^38^ No additional bands in the region around 1900 cm^−1^ appeared, which rules out the presence of bridge/hollow CO–Co vibrations, characteristic of larger Co ensembles.

### XAFS studies

**Fig. 6:**
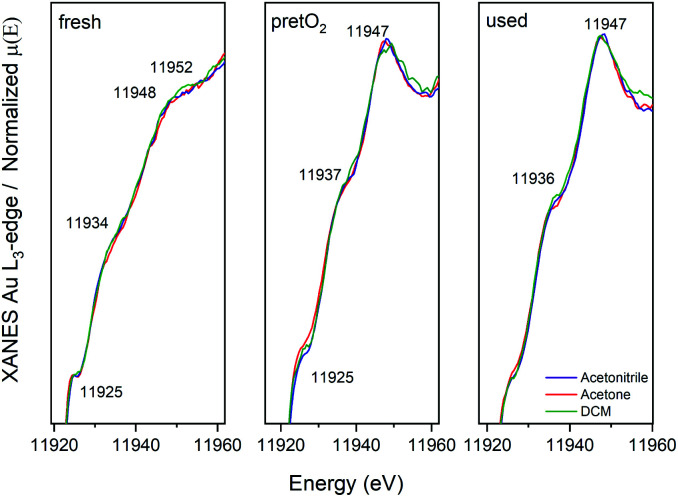
XANES spectra at the Au L_3_-edge of CoAu/CeO_2_ catalysts: as prepared, pretreated and after CO oxidation reaction.

Further details on the structure evolution of the CoAu nanocluster catalysts were explored by *in situ* XAFS studies at the Au L_3_-edge. Studies at the cobalt edge were not possible due to the low content. Fig. 6 displays the XANES spectra of the three catalysts as prepared (fresh), after the oxidative pretreatment (pretO_2_) and after CO oxidation (used).

After the pretreatment, a higher tendency towards the metallic state is observed, followed by a decrease and shift in the white line (≈11 925 eV). Moreover, changes in the peak around 11947 eV are visible. This is related to the decrease of Au–S bonds during pretreatment, resulting in an increase in the ratio of the Au–Au bonds leading to a higher metallic character. Further, almost no differences between the pretreated and used catalysts are observed in XANES in all cases.

**Fig. 7:**
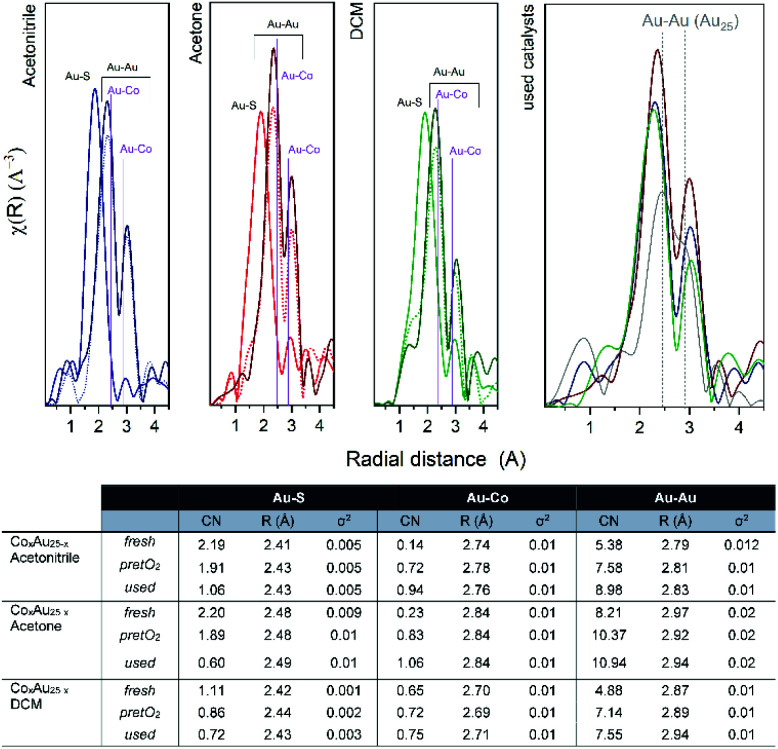
*R*-space EXAFS spectra and fitting results of CoAu/CeO_2_ catalysts.

The *R*-space and EXAFS fitting results of the bimetallic CoAu/CeO_2_ catalysts after each step are shown in Fig. 7. Slight differences between the pure clusters and the supported ones in CN (coordination number) and *R* (radial distance) values are observed, mainly in the Au–S bond which could be related to the strong interaction of the ligand shell with the CeO_2_ surface, already reported by previous studies.^39–41^ However, the Au cluster structure is preserved as no significant changes were obtained in the Au–Au CN. Moreover, the cluster structure is confirmed by the CN values, as they are significantly lower than those typical for fcc gold structures (CN = 12).^42–44^ As expected, the CN values of the Au–S bonds decrease after pretreatment, due to the removal of the ligand shell and this continues during the reaction. This is accompanied by an increase in Au–Co bonds. An initial location of some Co atoms at the staple positions, which migrate to the Au core surface during pretreatment and reaction, may explain this. In all cases, the Au–Au CN number is higher than that of Au–Co, confirming the good distribution of cobalt atoms within the Au cluster structure.

## Conclusions

Co–Au nanoalloys were prepared by doping Au_25_ nanoclusters, which resulted in different distributions of cobalt atoms in the cluster structure and thus in different properties. This was evident from their reactivity in the CO oxidation reaction, which showed an increase in catalytic activity when compared to the undoped system, related to the creation of Co and CoAu active sites. *In situ* spectroscopic studies revealed the evolution of the doped clusters to nanoalloys with well dispersed cobalt atoms within the Au cluster structure during pretreatment and reaction.

The present work opens new possibilities for the preparation of Co–Au nanoalloys in the cluster regime and for tuning their properties. In addition, these studies contribute to a better understanding of the structural dynamics of bimetallic nanoclusters that evolve into nanoalloys, which is crucial information for the future development and application of nanoalloy clusters.

## Author contributions

Synthesis of the Au nanoclusters was performed by J. O., S. R. and N. B.; kinetic measurements by S. R., infrared studies by M. M., XPS analysis and data evaluation by F. S. and C. R., mass spectrometry analysis attempts by H. D., XAFS evaluation by W. O. and data evaluation was done by N. B. Final interpretation and manuscript preparation was led by N. B. with contributions from all authors.

## Conflicts of interest

There are no conflicts to declare.

## Supplementary Material

FD-242-D2FD00120A-s001
