# Supplementary material for: Doped metal clusters as bimetallic AuCo nanocatalysts: insights into structural dynamics and correlation with catalytic activity by in situ spectroscopy
Source: Faraday Discuss. 2022 Jul 13;242:94–105. doi: 10.1039/d2fd00120a (PMC9890489; doi:10.1039/d2fd00120a)
Supplement: FD-242-D2FD00120A-s001 [file FD-242-D2FD00120A-s001.pdf]

Electronic Supplementary Material (ESI) for Faraday Discussions.
This journal is © The Royal Society of Chemistry 2022

Supporting Information

Doped metal clusters as bimetallic AuCo nanocatalysts: insights into structural dynamics and correlation with catalytic activity by in situ spectroscopy

Noelia Barrabés[a*], Jon Ostolaza[a,b], Sarah Reindl[a], Martin Mähr[a], Florian Schrenk[a], Hedda Drexler[a], Christoph Rameshan[a], Wojciech Olszewski[c], Günther Rupprechter[a]

[a] *Institute of Materials Chemistry, Technische Universität Wien, Getreidemarkt 9/165, 1060 Vienna, Austria*
[b]*Department of Chemical Engineering and Biotechnology, University of Cambridge, Philippa Fawcett Drive, Cambridge CB3 0AS, UK*
[c] *Faculty of Physics, University of Bialystok, ul. K. Ciolkowskiego 1L, 15-245 Bialystok, Poland*

corresponding author email: Noelia.rabanal@tuwien.ac.at

Experimental Details

Synthesis of $Co_xAu_{25-x}(SC_2H_4Ph)_{18}$. $CoAu_{25-x}(SC_2H_4Ph)_{18}$ was synthesized following a modified protocol from previous bimetallic nanoclusters synthesis.[1] Briefly, 58.6 mg $Co(NO_3)_2 \cdot 6H_2O$ (0.2 mmol) were dissolved in 25 ml THF together the gold salt $HAuCl_4 \cdot 3H_2O$ (0.8 mmol) was dissolved in 5 ml THF and added to the cobalt salt solution in a three-necked flask. The solution was stirred for 10 minutes before adding 654 mg of Tetraoctylammoniumbromide (TOAB) (4.2 mmol). After stirring until fully dissolved the reaction mixture was cooled down in an ice bath and covered with aluminium foil. After 40 minutes 0.67 ml of 2-phenylethanethiol (2-PET) (5 mmol) were added and stirred for 90 minutes. 381 mg of the strong reductant $NaBH_4$ (10 mmol) in ice cold water were rapidly added, leading to a black reaction mixture. After four hours of stirring, the solvent was evaporated. The black solids were filtrated, thoroughly washed with Methanol and centrifuged to separate them from thiolate ligand precursor and by-products. Subsequently, the nanoclusters were dissolved in DCM and filtered again, before the DCM was removed by rotary evaporation. To extract the different sized clusters, three extractions with first Acetonitrile, then Acetone and finally DCM were performed. Each extraction phase was then further separated by size exclusion chromatography column (SEC). The samples were characterized by UV-Vis spectroscopy and matrix-assisted laser desorption ionization (MALDI) mass spectrometry.

Synthesis of $Au_{25}(SC_2H_4Ph)_{18}$. The synthesis was carried out following a protocol by Shivare *et al.*[2] 50ml of THF and 500mg of $HAuCl_4 \cdot 3H_2O$ were mixed with 1.2eq. of TOAB and stirred for 10 min. Then, 0.85 ml of phenylethyl mercaptan was added to the solution and stirred until transparent. 480 mg of $NaBH_4$ in 10 ml of ice-cold water was added at once, leading to a dark brown reaction mixture. The solution was stirred for 4 days under ambient conditions, before the solvent was evaporated and the precipitate was washed several times with methanol. The clusters were then separated by Size Exclusion

Chromatography (SEC) and their purity evaluated by Ultraviolet-Visible (UV-Vis) spectroscopy and matrix-assisted laser desorption ionization (MALDI) mass spectrometry.

Characterization Techniques

UV-Vis spectra of nanoclusters dissolved in CH_2Cl_2 were recorded on a Perkin Elmer Lambda 750 UV-Vis spectrometer. Diffuse Reflectance Spectroscopy (DRS) of the nanoparticle catalysts was performed using the same instrument coupled to a 60 mm integration sphere.

All matrix-assisted laser desorption ionization (MALDI) mass spectrometric measurements were performed using a reflectron (RTOF) mass spectrometer (Shimadzu). For analytical experiments, 2,4,6-trihydroxyacetophenone (Sigma-Aldrich) was selected as MALDI-MS matrix. MALDI-RTOF mass spectra were acquired near threshold laser irradiance to obtain mass spectra of sufficient mass spectrometric resolution [3000–5000 at full width half-maximum (fwhm)]. All displayed mass spectra were based on averaging 300–600 single and unselected laser pulses (λ = 337 nm at 50 Hz).

UV-Vis spectra and MALDI mass spectra of the Au_{25} nanocluster sample in solution

Both the UV-Vis spectrum of $[Au_{25}(SC_2H_4Ph)_{18}]^-$ and the dominant peak at m/z = ≈7394 in the MALDI mass spectrum are in good agreement with the reported data.[2]

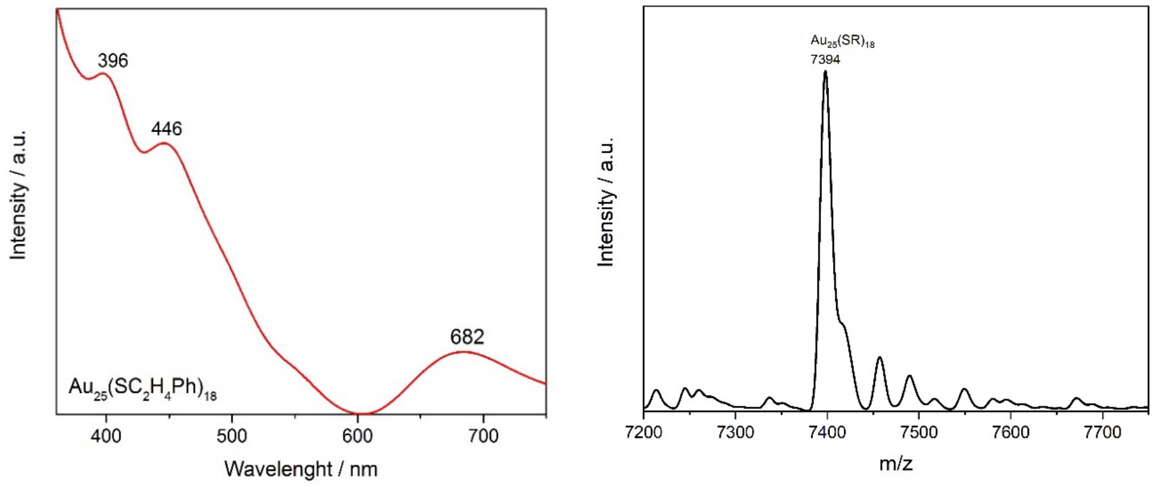

Figure S1. UV-Vis (left) and MALDI mass spectrum (right) of $Au_{25}(SC_2H_4Ph)_{18}$.

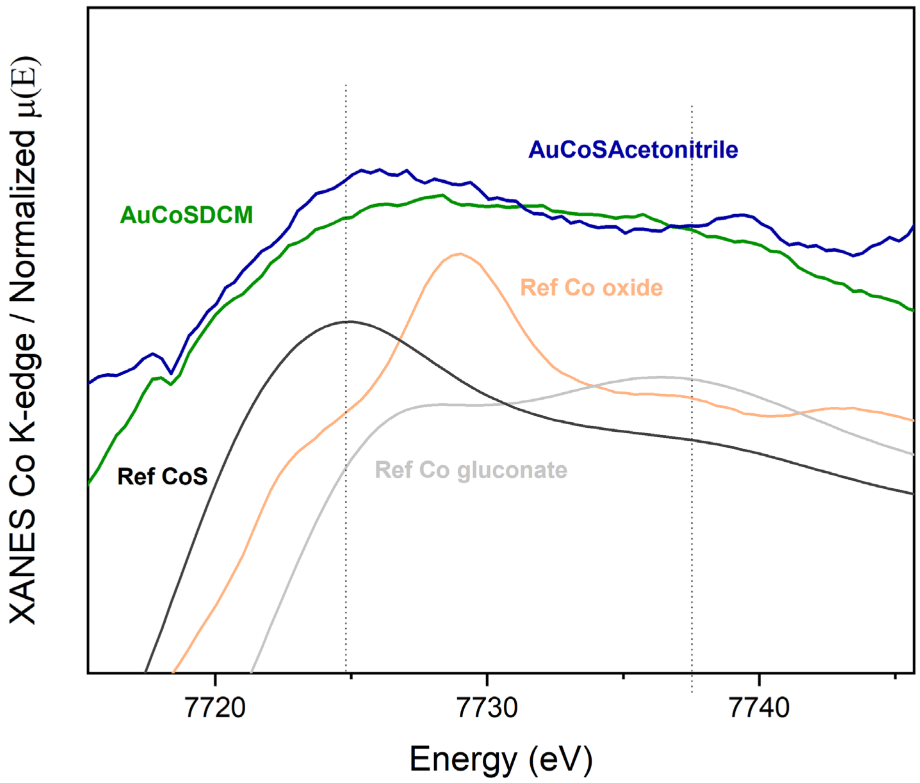

Figure S2. CO K-edge XANES of bimetallic cluster with reference materials

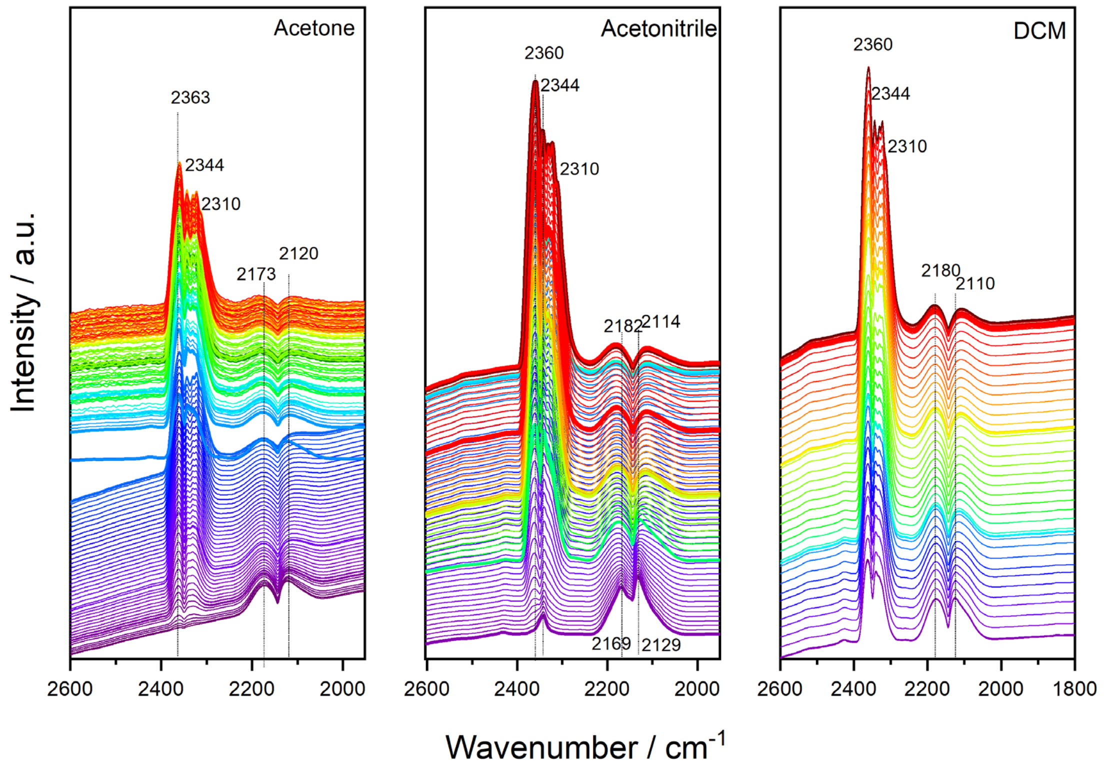

Figure S3. *In situ* IR spectra during CO oxidation reaction from room temperature to 250°C.

X-ray photoelectron spectroscopy (XPS) measurements of the spent catalysts were performed on a lab based in situ NAP-XPS system equipped with a Phoibos 150 NAP hemispherical analyzer and a XR 50 MF X-ray source (microfocus), all SPECS GmbH. Spectra were recorded with monochromatic Al Kα radiation and data were analysed with the CasaXPS software. Peaks were fitted after linear background subtraction with Gauss−Lorentz sum functions. The spectra were referenced to the Fermi edge and the C1s signal. Peak positions and full width at half-maximum (FWHM) were left unconstrained. Au4f peaks were fitted with 3.7 eV doublet separation and a fixed ratio of 4:3 for Au4f$^{7/2}$ and Au4f$^{5/2}$.

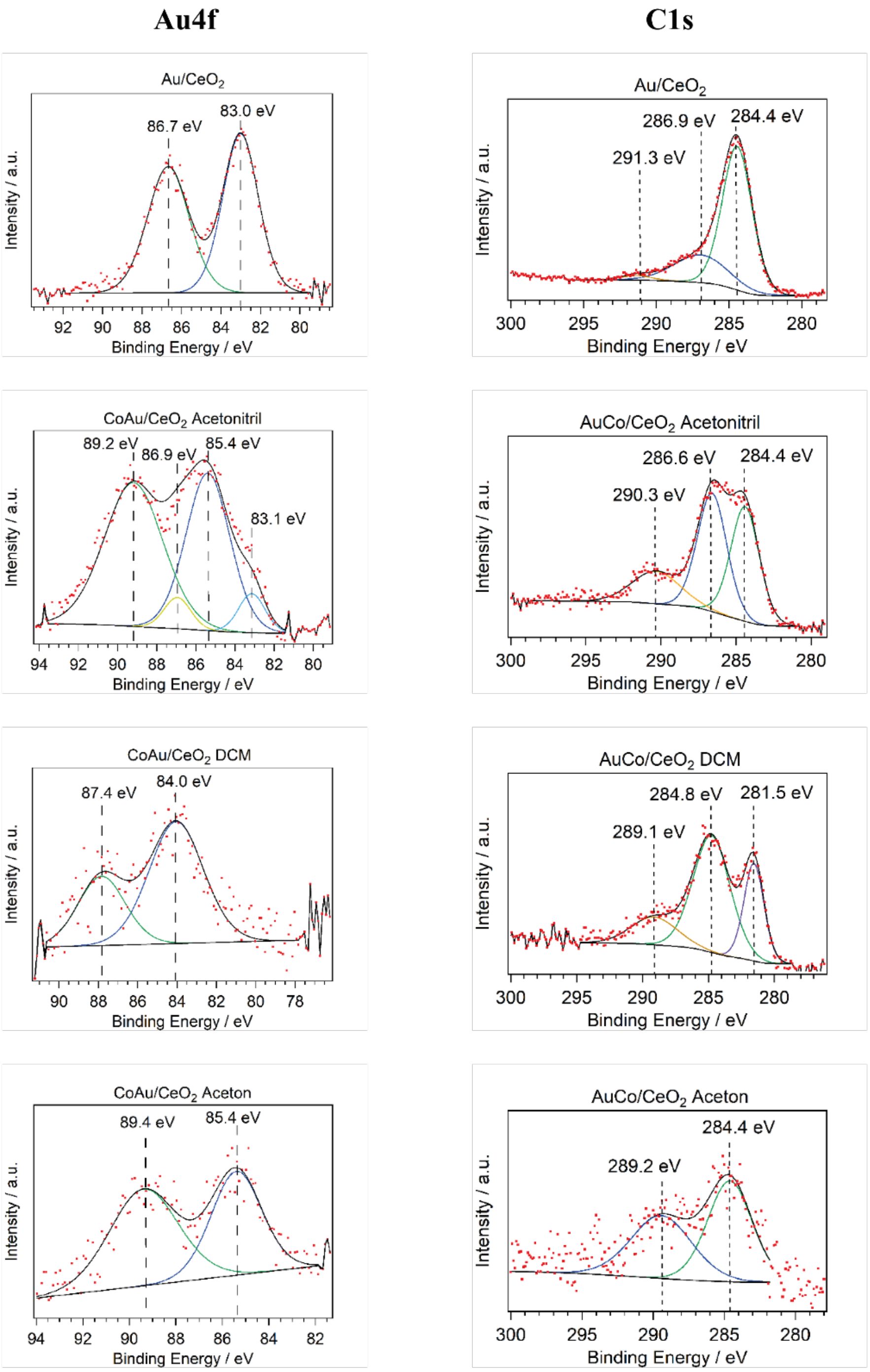

Figure S4. Fitted Au4f spectra (left) and C1s spectra (right) of the Au25/CeO2 sample as well as the samples extracted with Acetonitrile, DCM and Acetone.

The XPS analysis shows that the undoped reference sample (Au/CeO_2) has a binding energy for the Au4$f^{7/2}$ transition of 83.0 eV. This is about 1 eV lower than typical values. This behavior could be due to nanosize or charge transfer effects. In addition, only one Au species was found in this sample. The C1s transition shows that three different carbon species. The main species, with a binding energy of 284.4 eV, can be attributed to graphitic carbon,[3] which probably formed during the reaction. Two species with higher binding energies were found, one at 286.9 eV and one at 291.3 eV. The compound at 286.9 eV is most likely a carbon bound to an electronegative partner such as oxygen or sulfur.[4, 5] However, since no sulfur was present on the surface, a deposited carbo-oxide species is most likely. This species has a peak area of around 35 % of the main component. The species with the highest binding energy at 291.3 eV indicated a highly oxidized carbon species with a very electron deficient carbon. This species cannot be further defined without more thorough investigation. However, since this species is difficult to detect, having only about 3% of the peak area of graphitic carbon, we did not pursue it further.

For the AuCo/CeO_2 Acetonitrile, the Au4f spectra showed the presence of two different Au species. While the major species with a Au4$f^{7/2}$ binding energy of 85.4 eV was not observed by us, the minor species has a very similar Au4$f^{7/2}$ binding energy to the undoped reference sample (83.1 eV vs. 83.0 eV). The C1s spectra looks similar to the Au_{25}/CeO_2 sample. The two species with the lower binding energies have their maxima at 284.4 eV and 286.6 eV only divagating from the undoped sample by 0.3 eV for the species with the higher binding energy. However, the peak ratio has shifted as the second component has a peak area around 5 % bigger than that of the graphitic carbon. Again, a species with even higher binding energy was found at 290.3 eV. However, this component is also more pronounced and has a peak area of about 45% of the graphitic carbon. It is possible that this is adsorbed CO2 on CeO2 derived from the reaction.[6]

The AuCo/CeO_2 DCM sample has only one Au species. The Au4$f^{7/2}$ binding energy of 84.0 eV fits to the expected value for metallic Au.[7] The C1s spectra, however, reveals the presence of carbidic carbon on the surface with a binding energy of 281.5 eV. This species was not detected in the other samples. Graphitic carbon is also present here at 284.4 eV. In addition, a species with higher binding energy (289.1 eV) is present, accounting for about 30% of the peak area of graphitic carbon. This could indicate a carbonate species. No Co could be detected for the sample as well as for the other Co-doped sample, most likely due to the low loading of the samples.

The sample extracted with AuCo/CeO_2 Acetone shows the Au species found also in the Acetonitril sample with a binding energy for the Au4f7/2 of 85.4 eV. In contrast, the two minor species seen in the previous measurement could not be found in this sample. However, it should be noted that due to differential charging of the Aceton extracted sample in UHV conditions the measurement was performed under 1 mbar of N2. This led to a decrease in signal intensity and the minor species could therefore be not visible due to the worse signal to noise ratio. The C1s region is dominated by graphitic carbon at a binding energy of 284.4 eV. Another contribution to the spectra could be identified with a species at a binding energy at 289.2 eV which was also observed in other samples.

REFERENCES

1. Y. Negishi, K. Igarashi, K. Munakata, W. Ohgake and K. Nobusada, *Chem. Commun.*, 2012, **48**, 660-662.
2. A. Shivhare, S. J. Ambrose, H. Zhang, R. W. Purves and R. W. J. Scott, *Chem. Commun.*, 2013, **49**, 276-278.
3. R. Blume, D. Rosenthal, J.-P. Tessonnier, H. Li, A. Knop-Gericke and R. Schlögl, *ChemCatChem*, 2015, **7**, 2871-2881.
4. O. Björneholm, A. Nilsson, H. Tillborg, P. Bennich, A. Sandell, B. Hernnäs, C. Puglia and N. Mårtensson, *Surf Sci*, 1994, **315**, L983-L989.
5. U. Gelius, P. F. Hedén, J. Hedman, B. J. Lindberg, R. Manne, R. Nordberg, C. Nordling and K. Siegbahn, *Phys Scripta*, 1970, **2**, 70-80.
6. C. Yang, F. Bebensee, J. Chen, X. Yu, A. Nefedov and C. Wöll, *Chemphyschem*, 2017, **18**, 1874-1880.
7. X. Zhang, A. Shan, S. Duan, H. Zhao, R. Wang and W.-M. Lau, *RSC Advances*, 2019, **9**, 40811-40818.
